# Daily variation in sleep characteristics in individuals with and without post traumatic stress disorder

**DOI:** 10.1186/s12888-021-03282-3

**Published:** 2021-06-05

**Authors:** Quinn M. Biggs, Robert J. Ursano, Jing Wang, Gary H. Wynn, Rohul Amin, Carol S. Fullerton

**Affiliations:** 1 Center for the Study of Traumatic Stress (CSTS), Department of Psychiatry, Uniformed Services University of the Health Sciences (USUHS), 4301 Jones Bridge Road, Bethesda, MD 20814 USA; 2 Henry M. Jackson Foundation for the Advancement of Military Medicine (HJF), Bethesda, MD USA; 3 Walter Reed National Military Medical Center (WRNMMC), Bethesda, MD USA

**Keywords:** Sleep, Symptom assessment, Ecological momentary assessment, Military personnel

## Abstract

**Background:**

Sleep disturbances are common in individuals with post traumatic stress disorder (PTSD). However, little is known about how daily variation in sleep characteristics is related to PTSD. This study examined the night-to-night and weekday versus weekend variation in sleep duration, sleep quality, trouble falling asleep, and difficulty staying asleep in individuals with and without PTSD.

**Methods:**

Participants (*N* = 157; 80 with PTSD, 77 without PTSD) completed daily self-reports of their nighttime sleep characteristics for 15 consecutive days. Linear mixed models were used to examine the associations between the 7 days of the week and weekday versus weekend variation in sleep characteristics and PTSD.

**Results:**

Individuals with PTSD reported shorter sleep duration, lower sleep quality, more trouble falling asleep, and more difficulty staying asleep than individuals without PTSD. The pattern of change across the week and between weekdays and weekends was different between those with and without PTSD for sleep quality and trouble falling asleep. Among those with PTSD, sleep duration, sleep quality, and trouble falling asleep differed across the 7 days of the week and showed differences between weekdays and weekends. For those without PTSD, only sleep duration differed across the 7 days of the week and showed differences between weekdays and weekends. Neither group showed 7 days of the week nor weekday versus weekend differences in difficulty staying asleep.

**Conclusions:**

On average those with PTSD had shorter sleep duration, poorer sleep quality, and greater trouble falling and staying asleep. In particular, the day of week variation in sleep quality and trouble falling asleep specifically distinguishes those with PTSD from those without PTSD. Our findings suggest that clinical care might be improved by assessments of sleep patterns and disturbances across at least a week, including weekdays and weekends. Future studies should explore the mechanisms related to the patterns of sleep disturbance among those with PTSD.

**Supplementary Information:**

The online version contains supplementary material available at 10.1186/s12888-021-03282-3.

## Background

Sleep disturbances are common in individuals with post traumatic stress disorder (PTSD) [1]. Up to 92% of those with PTSD have reported at least one sleep disturbance in both general population and military samples [2–4]. Individuals with PTSD are more likely to have a shorter sleep duration [5] and report poor sleep quality [6] compared to those without PTSD. Having trouble falling asleep and difficulty staying asleep (the predominant symptoms of insomnia) are part of the diagnosis of PTSD [7] and are among the most frequently reported symptoms of post traumatic stress [8, 9]. Examining the day of week (DOW) patterns in sleep disturbances in those with and without PTSD may aid in understanding the fundamental mechanisms and possible treatments for PTSD.

Several studies have examined the relationship between sleep disturbances and next day PTSD symptoms in populations with PTSD [10–12]. Sleep duration, sleep quality, trouble falling asleep, and difficulty staying asleep were generally associated with next day PTSD symptoms [10–12]. These studies provide some evidence that variation in sleep disturbances across time was high among individuals with PTSD. For instance, 58 and 63% of the variance in sleep duration and sleep quality, respectively, were due to within-person change [11]. To our knowledge, no studies have specifically looked at DOW variation in sleep characteristics among individuals with PTSD.

Daily assessment of sleep across time (e.g., “How many hours of sleep did you get last night?”) as opposed to retrospective mean assessment of sleep across time (e.g., “During the past month, how many hours of sleep did you get at night?”), can provide information about how sleep changes over time. Methods of repeated experience sampling including ecological momentary assessments (EMA) and daily sleep diaries are well-suited to assessing symptom change over time [13, 14]. Examination of DOW variation in sleep and sleep disturbances is likely to better capture the impact of situational factors such as work schedules, threat cues, and lifestyle factors.

Differences in sleep have, in general, been examined in two ways, by looking at mean change over time and intraindividual variability. In this study, we focus on change over time of sleep characteristics and how the pattern of variation may distinguish PTSD from non-PTSD. Specifically, we examined variation in sleep disturbances across the 7 days of the week and in weekdays versus weekends in individuals with and without PTSD. Understanding the pattern of sleep characteristics across the week may aid in distinguishing diagnoses, improve our understanding of the mechanisms of disorder, inform development of treatment interventions, and suggest possible causes of variation such as weekday versus weekend lifestyle factors (e.g., work, leisure activities, social support). Therefore, in this paper we examined the DOW variation of sleep duration, sleep quality, trouble falling asleep, and difficulty staying asleep in individuals with and without PTSD. Sleep characteristics were assessed by daily self-report for 15 consecutive days. Linear mixed models were used to examine each of the four sleep characteristics across the 7 days of the week and for weekdays versus weekends. We anticipated that sleep characteristics would be worse (shorter sleep duration, poorer sleep quality, more trouble falling and staying asleep) among those with PTSD compared to those without PTSD, but had no expectations about the relationship between daily or weekday versus weekend variation in sleep characteristics and PTSD.

## Methods

### Participants

Participants were *N* = 183 current and former U.S. Service members. A total of *n* = 157 were included in data analyses, *n* = 14 did not return the daily assessments, *n* = 10 did not provide three or more daily assessments of sleep, *n* = 1 did not complete the assessment of probable PTSD in the pre-questionnaire, and *n* = 1 was removed as an outlier. This study was part of a larger data collection project examining post traumatic stress in U.S. military personnel. Methods common to the larger project have been reported in prior publications [10, 15] and are included in Supplement 1. The research was approved by the Institutional Review Board of Walter Reed National Military Medical Center and the Uniformed Services University of the Health Sciences in Bethesda, Maryland. Informed consent was obtained after the procedures were explained and participation was voluntary.

### Procedure and measures

#### Recruitment and enrollment screening

Participants were recruited from a military treatment facility and completed a 26-item screening questionnaire (see Supplement 1).

#### Assessment of PTSD

Once enrolled, we determined probable PTSD. Participants completed a 79-item assessment of exposure to traumatic events (see Supplement 1). All participants had at least one qualifying traumatic exposure. Participants also completed the 20-item PTSD Checklist for the Diagnostic and Statistical Manual of Mental Disorders-Fifth Edition (PCL-5; DSM-5) [16]. The PCL-5 assessed DSM-5 PTSD clusters B (intrusion: items 1–5), C (avoidance: items 6–7), D (negative cognitions/mood: items 8–14), and E (hyperarousal: items 15–20). Two of the 20 items are related to sleep disturbance (“Repeated, disturbing dreams of the stressful experience” and “Trouble falling or staying asleep”). Each item had four response choices, 0 (*Not at all*) to 4 (*Extremely*), and the symptom severity score range was 0–80. The PCL-5 has demonstrated good internal consistency (α = .96), test-retest reliability (r = .84), and convergent and discriminant validity in Service member populations [17]. A diagnosis of probable PTSD was made by considering each item rated 2 (*Moderately*) or higher as an endorsed symptom, then following the DSM-5 diagnostic criteria requiring one or more cluster A traumatic exposures, one or more items from clusters B and C, two or more items from clusters D and E, and a symptom severity score of 38 or higher [16]. Of the *N* = 157 participants, *n* = 80 met criteria for probable PTSD (hereafter referred to as those with PTSD) and *n* = 77 did not meet criteria for PTSD.

#### Daily assessments

For the following 15 days, participants completed four assessments per day using an EMA methodology, including an assessment of sleep characteristics on the first assessment of each day (see Supplement 1). The first 39 consecutive subjects (24.8%) completed daily assessments on paper questionnaires (Phase 1) and the next 118 subjects (75.2%) completed the same assessments on an Apple Inc. iPad 2 with a software application designed specifically for use in this study (Phase 2). The phase of the study was controlled for all analyses and was not a significant covariate. In total, *N* = 1994 sleep assessments were collected. Of those, *n* = 47 (2.4%) were dropped from data analysis because they were completed too early (*n* = 15), too late (*n* = 18), were missing the completion date or time (*n* = 13), or due to an irreconcilable error in electronic data (*n* = 1). Of the *N* = 1947 assessments included in the analyses, *n* = 1482 (76.1%) were completed within 0–2 h, *n* = 331 (17.0%) within 2–4 h, and *n* = 134 (6.9%) within 4–6 h of the 6-h assessment completion window. The overall adherence rate (i.e., percentage completed out of 2355 possible sleep assessments) was 82.7% in the present sample.

#### Sleep characteristics

Sleep duration was assessed with one item adapted from the Pittsburgh Sleep Quality Index (PSQI) [18], “How many hours of actual sleep did you get last night? (*This may be different than the number of hours you spent in bed*).” Participants wrote in the number of hours of sleep and, if necessary, responses were rounded to the nearest quarter hour (see Supplement 1 for details of the sleep measures). Sleep quality was assessed with one item adapted from the PSQI, “How would you rate your sleep quality overall last night?” Response choices ranged from 0 (*Very bad*) to 3 (*Very good*), with high scores reflecting better sleep quality. Trouble falling asleep was assessed with three items and difficulty staying asleep was assessed with six items, and these nine items were adapted from the PCL-5 [16], PSQI [18], SLEEP-50 [19] or developed for use in this study by the authors (RJU & CSF). Item instructions were “Below is a list of sleep problems. Please fill in the bubble according to what you experienced last night.” Response choices were 0 (*No*) and 1 (*Yes*). Cronbach’s alpha internal reliability, assessed at the person level, was acceptable or good for trouble falling asleep (α = 0.63) and difficulty staying asleep (α = 0.72). The mean of the items within the trouble falling asleep dimension and the difficulty staying asleep dimension was used in the analyses, indicating the percent of the sleep problem that the participant experienced during the previous night (range = 0–1), with high score reflecting more trouble falling asleep or more difficulty staying asleep.

#### DOW and weekday/weekend variables

A 7-day and a dichotomous weekday (Monday through Friday)/weekend (Saturday and Sunday) variable were created to test if the four sleep characteristics differed by the day of the week. The previous night’s sleep characteristics were reported on the first assessment of the following day (e.g., Saturday and Sunday’s report captured the sleep characteristics of Friday and Saturday night’s sleep).

### Data analyses

DOW variation in sleep characteristics was assessed using linear mixed models with daily assessments (Level-1) nested within subjects (Level-2). The first-order autoregression assumption (AR [1]) was compared to compound symmetry and was selected for within-subject residuals for all analyses (see Supplement 2 for further details). The intraclass correlation coefficient (ICC) was provided to identify the proportion of variance due to Level 1 and Level 2 in the analysis (see Supplement 2).

We first examined whether each sleep characteristic varied across the 7 days of the week or between weekdays versus weekends in the total sample and in individuals with and without PTSD. The total group analyses allowed us to examine sleep characteristic differences by PTSD group controlling for DOW and demographic covariates. The stratified analyses explored the DOW variation in individuals with PTSD, our group of primary interest, and provided a comparison to those without PTSD. We then examined whether participants with and without PTSD differed in each sleep characteristic across the 7 days of the week or between weekdays versus weekends by testing the interaction of PTSD group and DOW. Due to the low statistical power to detect cross-level interaction effects in mixed models [20, 21], we were aware that the interaction analysis was informative but limited. In the 7 DOW analyses the Tukey-Kramer method was used to adjust for multiple pairwise comparisons. Sex, age, race, education, and phase of the study (paper versus electronic) were included as covariates. Mixed model analyses were conducted in PC SAS version 9.3 (SAS Institute, Cary, North Carolina).

To determine the specific statistical power of our sample to detect the DOW difference as a within-subject predictor and PTSD group difference as a between-subject predictor, a series of Monte Carlo simulation post-hoc power analyses were conducted [22, 23]. Results showed a high statistical power to detect a significant within-subject predictor, but a relatively low power for a between-subject predictor. For example, for sleep duration, at a power of 80% with 77 individuals (the smaller group size in the current study) and a mean of 12 observations per individual, we are able to detect a weekday versus weekend difference of .23 standard deviations, which can be considered a small effect size. A significant between-subject predictor (e.g., PTSD group) in an analysis of 157 individuals would require an effect size of .43 standard deviations, which can be considered a medium effect size. Post-hoc power analyses were conducted in Mplus version 7.11 [24].

## Results

### Sample demographics and descriptive statistics

Demographics and descriptive statistics are shown in Table 1. Mean age of participants in the analytic sample (*N* = 157) was 41.50 (range 19–76). The majority (59.2%, *n* = 93) were male, White (65.0%, *n* = 102), and 55.4% (*n* = 87) had a bachelor’s degree education or higher. The majority (65.6%, *n* = 103) were married with 76.4% (*n* = 81) currently living with their spouse. In total, 80 (51.0%) had PTSD and 77 (49.0%) did not have PTSD. There were no differences between those with PTSD and those without PTSD on demographic characteristics, except that individuals with PTSD were younger than those without PTSD (38.84 vs. 44.34, *t* = 2.54, *p* = .012). Comparing individuals with and without PTSD by person means, those with PTSD reported significantly shorter sleep duration (5.33 vs. 5.98, *t* = 3.20, *p* = .002), lower sleep quality (1.40 vs. 1.64, *t* = 3.09, *p* = .002), more trouble falling asleep (0.54 vs. 0.25, *t* = − 6.52, *p* < .001), and more difficulty staying asleep (0.35 vs. 0.18, *t* = − 5.98, *p* < .001).

**Table 1:** Demographic Characteristics and Descriptive Statistics of the Total Sample and Participants with and without PTSD

Categorical	Total sample (*N* = 157)	With PTSD (*n* = 80)	Without PTSD (*n* = 77)	*Χ*^*2*^	*p*
	*n* (%)	*n* (%)	*n* (%)		
Sex				1.21	.271
Male	93 (59.2)	44 (55.0)	49 (63.6)		
Female	64 (40.8)	36 (45.0)	28 (36.4)		
Race				0.00	.993
White	102 (65.0)	52 (65.0)	50 (64.9)		
Non-white	55 (35.0)	28 (35.0)	27 (35.1)		
Education				2.85^a^	.092
High school or G.E.D.	7 (4.5)	5 (6.3)	2 (2.6)		
Some college/tech. School	63 (40.1)	34 (42.5)	29 (37.7)		
Bachelor’s degree	27 (17.2)	16 (20.0)	11 (14.3)		
Graduate degree	60 (38.2)	25 (31.3)	35 (45.5)		
Marital Status				0.25	.618
Currently married	103 (65.6)	51 (63.8)	52 (67.5)		
Not currently married	54 (34.4)	29 (36.3)	25 (32.5)		
Living with spouse (married)				1.31	.253
Yes	81 (76.4)	38 (71.7)	43 (81.1)		
No	25 (23.6)	15 (28.3)	10 (18.9)		
Continuous (range)	*M* (*SD*)	*M* (*SD*)	*M* (*SD*)	*t*	*p*
Age (19–76)	41.5 (13.7)	38.8 (11.8)	44.3 (15.0)	2.54	.012
Sleep duration (1.00–9.13)	5.6 (1.3)	5.3 (1.3)	6.0 (1.2)	3.20	.002^b^
Sleep quality (0–3)	1.5 (0.5)	1.4 (0.5)	1.6 (0.5)	3.09	.002^b^
Trouble falling asleep (0–1)	0.4 (0.3)	0.5 (0.3)	0.2 (0.2)	−6.52	<.001^b^
Difficulty staying asleep (0–1)	0.3 (0.2)	0.3 (0.2)	0.2 (0.1)	−5.98	<.001^b^

*Note*. PTSD = post traumatic stress disorder. ^a^Mantel-Haenszel Chi-Square was conducted for a linear association between education and PTSD group. ^b^Using person means, results of *t*-test statistics showed that individuals with PTSD reported significantly shorter sleep duration, lower sleep quality, more trouble falling asleep, and more difficulty staying asleep

### Intraclass correlation coefficients

The ICCs (the proportion of variance associated with between-person differences) among the total sample, individuals with PTSD, and individuals without PTSD were as follows: 38.4, 37.2, and 36.2% of the total variance in sleep duration; 27.2, 22.0, and 30.5% in sleep quality; 61.4, 55.8, and 52.1% in trouble falling asleep; and 47.9, 45.5, and 36.4% in difficulty staying asleep, respectively (see Supplement 2). The remaining proportion of the variances (38.6–78.0%) were due to the within-person variance in each sleep characteristic.

### DOW variation (total sample, with PTSD, and without PTSD)

Sleep duration, sleep quality, and trouble falling asleep varied across the 7 days in the total sample and in individuals with PTSD (total sample: *F*(6, 862) = 11.62, *p* < .001, *F*(6, 863) = 3.54, *p* = .002, *F*(6, 863) = 3.01, *p* = .007; with PTSD: *F*(6, 431) = 5.08, *p* < .001, *F*(6, 431) = 3.30, *p* = .004, *F*(6, 431) = 2.95, *p* = .008; see Supplement 3). In individuals without PTSD, only sleep duration varied across the 7 days (*F*(6, 425) = 6.89, *p* < .001). Difficulty staying asleep did not vary across the 7 days or between weekdays and weekends for the total sample or those with or without PTSD. As results are similar for the total sample and individuals with PTSD (see Supplement 3), the descriptions that follow only provide details for individuals with and without PTSD.

#### Sleep duration

**Fig. 1:**
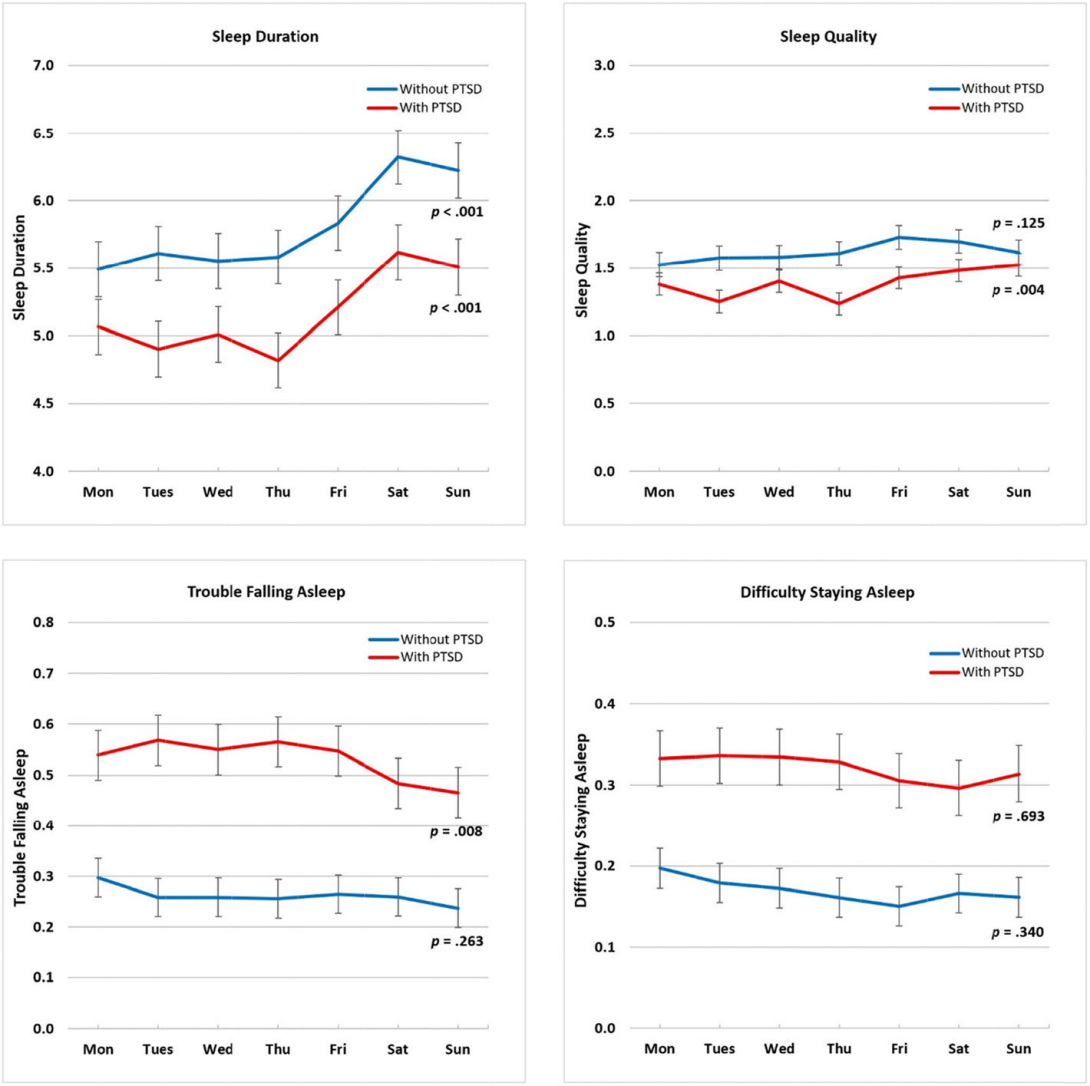
DOW Variation in Sleep Characteristics by Day of Report in Participants with and without PTSD. *Note*. The previous night’s sleep characteristics were reported on the first assessment of the following day (e.g., Monday’s report captured the sleep characteristics of Sunday night’s sleep). The *p*-value was the result of testing whether there was a difference in the sleep characteristic across the 7 days

**Fig. 2:**
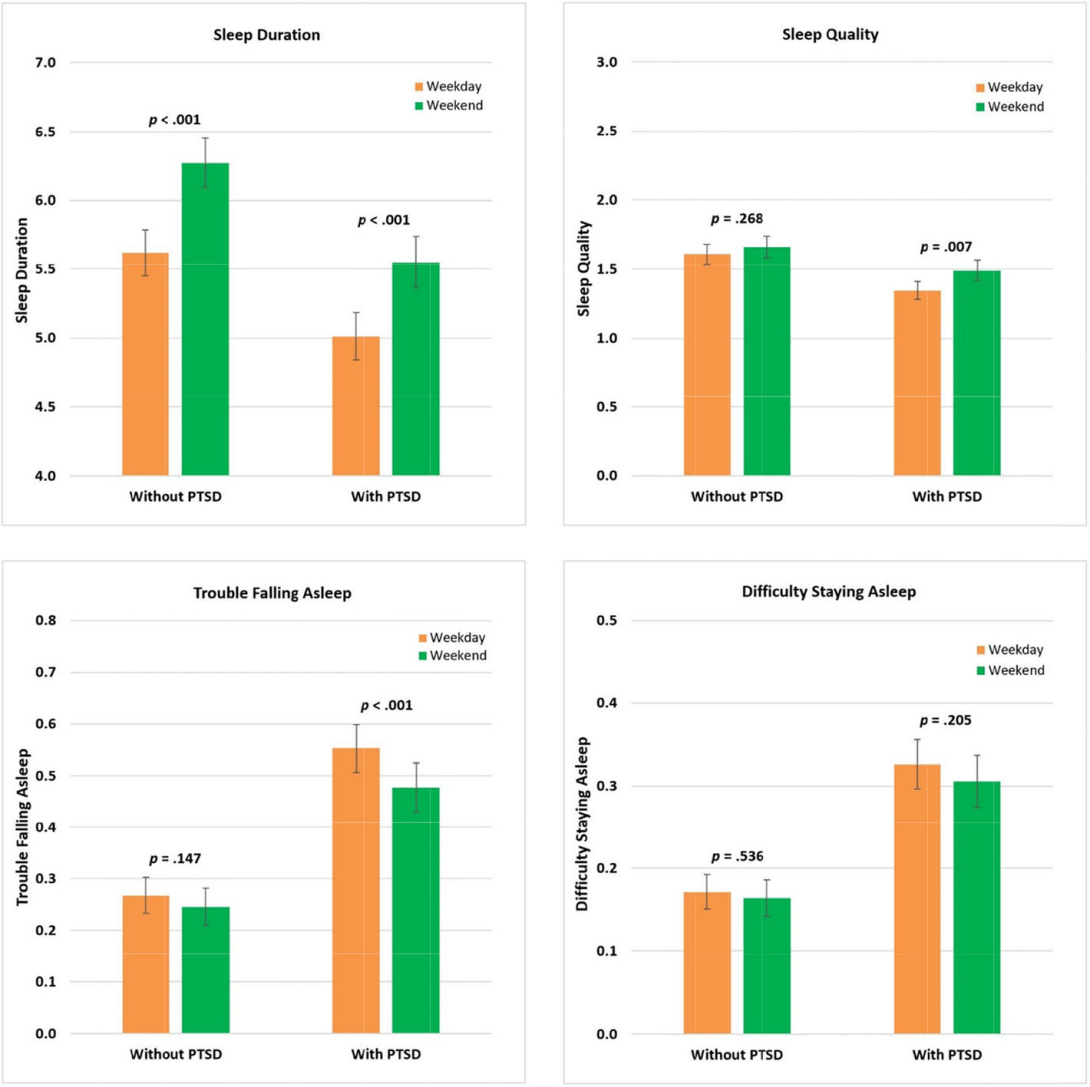
Weekday-Weekend Variation in Sleep Characteristics by Day of Report in Participants with and without PTSD. *Note*. The previous night’s sleep characteristics were reported on the first assessment of the following day. Weekday included the previous night’s sleep reported on Monday through Friday and Weekend included the previous night’s sleep reported on Saturday and Sunday. The *p*-value was the result of testing whether there was a difference in the sleep characteristic between weekdays and weekends

In individuals with and without PTSD, each of the five weekdays (Monday to Friday) had shorter sleep duration than Saturday, which was set as the reference (see Fig. 1 and Supplement 3). There was no difference between Saturday and Sunday. Pairwise comparisons with Tukey-Kramer adjustment further showed that in both analytic samples Saturday was different from Monday, Tuesday, Wednesday, and Thursday, and Sunday was different from Tuesday and Thursday. In addition, among those without PTSD, Sunday was different from Monday and Wednesday. By weekday (Monday through Friday) versus weekend (Saturday and Sunday), the Least-squares estimated means of sleep duration among individuals with PTSD were 5.01 for weekdays and 5.55 for weekends, β = − 0.60 [− 0.76, − 0.45], *p* < .001 (see Table 2, Table 3, and Fig. 2). Among individuals without PTSD, the estimated means of sleep duration were 5.61 for weekdays and 6.27 for weekends, β = − 0.66 [− 0.87, − 0.44], *p* < .001.

**Table 2:** Weekday versus Weekend Differences in Sleep Duration in the Total Sample and Participants with and without PTSD: Results of Mixed Models

Parameter	Total sample (*N* = 157)		With PTSD (*N* = 80)		Without PTSD (*N* = 77)	
	Coefficient [95% CI]	*p*	Coefficient [95% CI]	*p*	Coefficient [95% CI]	*p*
Fixed effects						
Intercept	7.04 [6.63, 7.44]	<.001	6.44 [5.93, 6.95]	<.001	7.04 [6.51, 7.57]	<.001
Female vs. male	−0.05 [−0.42, 0.32]	.789	−0.04 [− 0.57, 0.49]	.883	− 0.09 [− 0.66, 0.48]	.745
Age^a^	0.00 [− 0.01, 0.02]	.864	0.00 [− 0.03, 0.03]	.903	0.00 [− 0.02, 0.02]	.794
White vs. non-white	− 0.92 [−1.32, − 0.52]	<.001	−0.84 [−1.43, − 0.26]	.005	−1.00 [− 1.58, − 0.43]	<.001
Some college or lower vs. bachelor’s or higher^b^	−0.64 [− 1.07, − 0.20]	.004	−0.65 [− 1.32, 0.01]	.055	− 0.57 [− 1.20, 0.06]	.077
Phase 1 vs. 2	−0.06 [− 0.50, 0.38]	.778	−0.24 [− 0.89, 0.41]	.465	0.12 [− 0.51, 0.75]	.712
Weekday vs. weekend	−0.60 [− 0.76, − 0.45]	<.001	−0.54 [− 0.76, − 0.32]	<.001	−0.66 [− 0.87, − 0.44]	<.001
PTSD vs. non-PTSD	−0.56 [− 0.93, − 0.20]	.003	–	–	–	–

*Note*. CI = confidence interval; PTSD = post traumatic stress disorder. ^a^Age was centered at the group mean. ^b^Bachelor’s degree or higher was set as the reference. See Supplement 4a-4c for results of mixed models for sleep quality, trouble falling asleep, and difficulty staying asleep

**Table 3:** Model Regression Coefficients Testing Sleep Characteristic Differences: Weekday/Weekend and with/without PTSD in the Total Sample and in those with PTSD and without PTSD

	Total sample (*N* = 157)		With PTSD (*n* = 80)		Without PTSD (*n* = 77)	
	Coefficient [95% CI]	*p*	Coefficient [95% CI]	*p*	Coefficient [95% CI]	*p*
Sleep duration						
Weekday vs. weekend	−0.60 [− 0.76, − 0.45]	<.001	−0.54 [− 0.76, − 0.32]	<.001	−0.66 [− 0.87, − 0.44]	<.001
PTSD vs. non-PTSD	−0.56 [− 0.93, − 0.20]	.003	–	–	–	–
Sleep quality						
Weekday vs. weekend	−0.10 [− 0.17, − 0.03]	.004	−0.15 [− 0.25, − 0.04]	.007	−0.05 [− 0.15, 0.04]	.268
PTSD vs. non-PTSD	−0.19 [− 0.34, − 0.04]	.012	–	–	–	–
Trouble falling asleep						
Weekday vs. weekend	0.05 [0.02, 0.07]	<.001	0.07 [0.04, 0.11]	<.001	0.02 [−0.01, 0.05]	.147
PTSD vs. non-PTSD	0.28 [0.19, 0.37]	<.001	–	–	–	–
Difficulty staying asleep						
Weekday vs. weekend	0.01 [−0.01, 0.03]	.156	0.02 [−0.01, 0.05]	.205	0.01 [−0.02, 0.03]	.536
PTSD vs. non-PTSD	0.16 [0.10, 0.22]	<.001	–	–	–	–

*Note*. CI = confidence interval; PTSD = post traumatic stress disorder. Separate analyses were conducted for each sleep characteristic. Covariates in each model included sex, age, race, education, and phase of the study

#### Sleep quality

In individuals without PTSD, there was no difference in sleep quality across the 7 days or between weekdays and weekends. In individuals with PTSD, sleep quality on Tuesday and Thursday was worse than Saturday, which was set as the reference (see Fig. 1 and Supplement 3). Pairwise comparisons with Tukey-Kramer adjustment showed that Saturday was different from Thursday, and Sunday was different from Tuesday and Thursday. By weekday versus weekend, the Least-squares estimated means of sleep quality in individuals with PTSD were 1.34 for weekdays and 1.49 for weekends, β = − 0.15 [− 0.25, − 0.04], *p* = .007 (see Table 3, Fig. 2, and Supplement 4a).

#### Trouble falling asleep

In individuals without PTSD, there was no difference in trouble falling asleep across the 7 days or between weekdays and weekends. In individuals with PTSD, more trouble falling asleep was reported on Tuesday, Wednesday, Thursday, and Friday than Saturday, the reference (see Fig. 1 and Supplement 3). Pairwise comparisons with Tukey-Kramer adjustment showed that Sunday was different from Tuesday and Thursday. By weekday versus weekend, the Least-squares estimated means of trouble falling asleep in individuals with PTSD were 0.55 for weekdays and 0.48 for weekends, β = 0.07 [0.04, 0.11], *p* < .001 (see Table 3, Fig. 2, and Supplement 4b).

#### Difficulty staying asleep

In individuals with and without PTSD, there was no difference in difficulty staying asleep across the 7 days or between weekdays and weekends (see Table 3, Fig. 2, and Supplement 4c).

### Differences between individuals with and without PTSD

There were significant differences by PTSD group in all four sleep characteristics after controlling for weekday versus weekend (similar results were found for the 7-day DOW) and demographic covariates (see Table 3). Individuals with PTSD, compared to individuals without PTSD, reported shorter sleep duration (Least-squares estimated means = 5.34 vs. 5.90, β = − 0.56 [95% CI: − 0.93, −.20], *p* < .001), worse sleep quality (1.43 vs. 1.62, β = − 0.19 [− 0.34, − 0.04], *p* = .012), more trouble falling asleep (0.52 vs. 0.25, β = 0.28 [0.19, 0.37], *p* < .001), and more difficulty staying asleep (0.32 vs. 0.16, β = 0.16 [0.10, 0.22], *p* < .001).

### Interaction of PTSD group and DOW

The interaction of PTSD group and DOW examined whether participants with and without PTSD statistically differed in each sleep characteristic across the 7 days or between weekdays versus weekends. The only significant result was for the interaction of PTSD group and weekdays versus weekends on trouble falling asleep, *F*(1, 149) = 4.62, *p* = .033.

## Discussion

This study examined sleep characteristics (sleep duration, sleep quality, trouble falling asleep, and difficulty staying asleep) and their variation across the 7 days of the week and specifically for weekdays versus weekends in individuals with and without PTSD. Similar to others [5, 6, 8, 9], we found that on average those with PTSD had shorter sleep duration, poorer quality of sleep, and greater trouble falling and staying asleep. Although only two of these characteristics (i.e., trouble falling and staying asleep) are noted in the DSM-5 diagnosis of PTSD, in fact all four sleep characteristics differ between those with PTSD and those without PTSD. Importantly, the pattern of change across the 7 days of the week and the change between weekdays and weekends are different for those with PTSD compared to those without PTSD for sleep quality and trouble falling asleep. Among those with PTSD, sleep duration, sleep quality, and trouble falling asleep differed across the 7 days of the week and showed differences between weekdays and weekends. In contrast, for those without PTSD, only sleep duration differed across the 7 days of the week and showed differences between weekdays and weekends. For difficulty staying asleep, neither group differed across the 7 days of the week nor weekdays versus weekends.

Sleep duration varied across the 7 days of the week and specifically for weekdays versus weekends for both those with and without PTSD, which is consistent with previous studies [25–27]. For healthy adults, the average sleep duration is 7–8 h per night and duration is approximately 30 min longer on weekends compared to weekdays [25–27]. In our study, mean sleep duration was 5.34 h among those with PTSD and 5.90 h among those without PTSD, and both groups slept longer on weekends (approximately 32 min longer for those with PTSD and 40 min longer for those without PTSD). However, the average sleep duration of those with PTSD, even with more sleep on weekends, was never as high as the average weekday sleep duration of those without PTSD. Other studies have found that days in which sleep duration is much shorter or longer that an individual’s average are associated with worse cognitive functioning [28]. Habitual short sleep duration (e.g., < 5 h per night) and long sleep duration (e.g., > 9 h per night) are associated with increased risk for all-cause mortality [29–31]. Others have found that variation in the pattern between weekdays and weekends may also be associated with depressive symptoms [32], although not increased mortality [29].

Sleep quality varied across the 7 days of the week and was better on weekends than weekdays, but only among those with PTSD. Sleep quality is closely related to the other sleep characteristics [10]. The specific weekday versus weekend difference among those with PTSD may therefore suggest that PTSD-related stressors (such as threat cues) may be more common during weekdays or, alternatively, protective factors such as social support may be more present on weekends. Further study of sleep quality and its relationship to threat cues and social support is needed to understand the differences in sleep quality among those with and without PTSD.

Trouble falling asleep, like sleep quality, varied across the 7 days of the week and differed between weekdays and weekends, but only for those with PTSD. Falling asleep has been closely studied to identify behavioral and pharmacologic interventions to decrease sleep latency [33–35]. However, little is known about the neurobiology of falling asleep. PTSD is associated with being hyperalert to cues of threat which may also interfere with falling asleep. Further study of the neurobiological and behavioral aspects of trouble falling asleep among those with PTSD may aid in identifying interventions for this sleep characteristic and improve our understanding of the neurobiology of PTSD.

It is valuable to note, as above, that sleep quality and trouble falling asleep showed weekday-weekend variation only among those with PTSD. These sleep characteristics may therefore offer unique opportunities to understand PTSD as well as important targets for intervention. Weekday-weekend variation in sleep disturbances may be related to a number of factors expected to differ between those with and without PTSD including occupational stress, daily activities, social interactions, substance use, as well as fundamental neurobiological differences.

Difficulty staying asleep, in contrast to the other three sleep characteristics, did not show variation across the 7 days of the week nor weekdays versus weekends in either group. However, those with PTSD reported significantly more frequent difficulty staying asleep (32% of nights versus 16% of nights among those without PTSD). Prior research has shown that difficulty staying asleep was the strongest predictor of next day’s post traumatic stress symptoms [10]. These data therefore suggest that better understanding the greater frequency of difficulty staying asleep, rather than its temporal pattern may aid in understanding its relationship to next day’s post traumatic stress symptoms.

Our findings suggest that clinical care might be improved by assessments of sleep patterns and disturbances across at least a week, including weekdays and weekends. This is consistent with other studies that found one or two nights of sleep measurement may not accurately represent an individual’s habitual sleep pattern [36, 37]. Wide fluctuations in sleep characteristics among those with PTSD comprise a major challenge for clinicians, whose ministrations may be difficult to assess in light of such variations [38]. Sleep diaries can be an important addition to sleep assessments as they provide night-to-night information about sleep characteristics, promote discussions about sleep-related behavior, and may aid in identifying temporal relationships between sleep disturbances and daily events, which may be critical for interventions for sleep and PTSD. We know little about the importance of timing of interventions for maximum efficacy of behavioral or pharmacologic interventions. For instance, a medication to reduce trouble falling asleep may appear more or less effective depending on the day of the week that treatment is initiated. The individual’s sleep duration pattern may inform the choice of sleep medication and use of medication may not be necessary on nights when there is typically little trouble falling asleep.

The current study has several limitations. The measures are self-report and therefore subject to recall errors. This limitation is partially mitigated by collection of the daily report of sleep characteristics within hours of awakening and use of well-validated self-assessment measures. Sleep quality was measured with one item and results may have been different with a more extensive measure of sleep quality. The prevalence of sleep problems may be biased due to missing data. In addition, our sample consisted of current and former Service members and our findings may not generalize to civilians. Lastly, the statistical power to detect a between-person predictor and especially a significant interaction involving between-group predictors is limited. Thus, the results of interaction analyses are considered exploratory and only informative.

These results highlight the differences in sleep characteristics and their variation during the week between those with PTSD and without PTSD. Importantly, the pattern of sleep quality and trouble falling asleep specifically distinguishes those with PTSD from those without PTSD. Future studies should explore the mechanisms related to the patterns of sleep disturbance among those with PTSD and the factors associated with the substantial proportion of within-person variance in sleep disturbances. Further understanding of the neurobiology of sleep characteristics may aid in identifying the disruptions associated with PTSD and may help to identify potential targets for behavioral and pharmacologic interventions.

## Conclusions

On average those with PTSD had shorter sleep duration, poorer sleep quality, and greater trouble falling and staying asleep. Importantly, the pattern of change across the 7 days of the week and between weekdays and weekends are different for those with PTSD compared to those without PTSD for sleep quality and trouble falling asleep. Our findings suggest that clinical care might be improved by assessments of sleep patterns and disturbances across at least a week, including weekdays and weekends. Future studies should explore the mechanisms related to the patterns of sleep disturbance among those with PTSD.

## Supplementary Information

**Additional file 1.** Supplements 1-4c.

## Data Availability

The datasets used and/or analyzed during the current study are available from the corresponding author on reasonable request.

## Abbreviations

AR(1): First-order autoregression assumption
CS: Compound symmetry
DOW: Day of week
DSM-5: Diagnostic and Statistical Manual of Mental Disorders-Fifth Edition
EMA: Ecological momentary assessment
ICC: Intraclass correlation coefficient
PCL-5: PTSD Checklist for the DSM-5
PSQI: Pittsburg Sleep Quality Index
PTSD: Post traumatic stress disorder
