# Supplementary material for: Daily variation in sleep characteristics in individuals with and without post traumatic stress disorder
Source: BMC Psychiatry. 2021 Jun 5;21:292. doi: 10.1186/s12888-021-03282-3 (PMC8180043; doi:10.1186/s12888-021-03282-3)
Supplement: Supplementary file 1 — Additional file 1. Supplements 1-4c. [file 12888_2021_3282_MOESM1_ESM.docx]

**Supplement 1.** Methods Common to the Larger Data Collection Project

The descriptions of the methods in this supplement are common to the larger data collection project and have been previously reported [1, 2].

**Procedure and measures**

***Recruitment and enrollment screening***

Service members self-referred from advertisements or approach to a recruiting table. The advertisements and recruiting personnel stated that the study was seeking to enroll individuals with post traumatic stress symptoms. Participation in the study was independent of medical records and any mental or medical healthcare the Service member may have been receiving. Inclusion criteria included being 18 years of age or older, fluent in English, and from a uniformed service (Air Force, Army, Army National Guard, Army Reserves, Coast Guard, Marines, Navy, and U.S. Public Health Service). Exclusion criteria included suicidal or homicidal behavior in the past three months or diagnosis of or care for a psychotic disorder in the past year. Service members meeting the inclusion and exclusion criteria were administered a screening questionnaire to assess for common symptoms after a “stressful experience” using an aggregate of 26 symptoms of post traumatic stress disorder (PTSD), depression, and generalized anxiety. PTSD symptoms were measured by 18 non-sleep items from the 20-item PTSD Checklist for the Diagnostic and Statistical Manual of Mental Disorders-Fifth Edition (PCL-5; DSM-5) [3]. Depression symptoms were measured by 6 items from the 9-item Patient Health Questionnaire Depression Scale (PHQ-9) [4, 5]. Generalized anxiety symptoms were measured by 2 items from the 7-item Generalized Anxiety Disorder-7 (GAD-7) [6]. The response format of the 26 items was modified to an 11-point scale, 0 (*not at all*) to 10 (*extremely*), (range 0-260) and the timing was “…over the past month.” Service members with a score of 40 or more were enrolled in the study.

***Assessment of exposure to traumatic events***

After enrollment, participants completed an assessment of exposure to traumatic events, which included 79 items that were adapted from multiple sources or developed for use in this study. Response choices varied by item but any response indicating a traumatic event had occurred at least once in the individual’s life was considered endorsement of that item.

Traumatic Event Exposure Items

| Childhood Experiences | |
| --- | --- |
| 1. | You were beaten up, had things stolen from you, or were terrorized by bullies at school or in the neighborhood^a^ |
| 2. | You were physically abused at home^a,b^ |
| 3. | You were sexually abused at home^a,b^ |
| 4. | Someone touched you or made you touch them in a sexual way against your will^a,b^ |
| 5. | Someone in your family hit you so hard that it left bruises or marks^a,b^ |
| 6. | Your parents (or the people who raised you) hit each other or beat each other up^a,c^ |
| 7. | You witnessed or experienced a violent crime, like a shooting or a rape^d^ |
|  |  |
| PTSD Diagnosis Criterion A Stressful Experiences | |
| 8. | Have you ever been in danger of being killed or injured prior to being in the military^e^ |
| 9. | Have you ever been in danger of being killed or injured during military service^e^ |
| 10. | Have you ever been in danger of being killed or injured since military service^e^ |
| 11. | Have you ever witnessed someone in danger of being killed or injured prior to being in the military^e^ |
| 12. | Have you ever witnessed someone in danger of being killed or injured during military service^e^ |
| 13. | Have you ever witnessed someone in danger of being killed or injured since military service^e^ |
|  |  |
| Life Events Checklist (Endorsed if response was *Happened to me* or *Witnessed it*) | |
| 14. | Natural disaster (for example, flood, hurricane, tornado, earthquake)^f,g^ |
| 15. | Fire or explosion^f,g^ |
| 16. | Transportation accident (for example, car accident, boat accident, train wreck, plane crash)^f,g^ |
| 17. | Serious accident at work, home, or during recreational activity^f,g^ |
| 18. | Exposure to toxic substance (for example, dangerous chemicals, radiation)^f,g^ |
| 19. | Physical assault (for example, being attacked, hit, slapped, kicked, beaten up)^f,g^ |
| 20. | Assault with a weapon (for example, being shot, stabbed, threatened with a knife, gun, bomb)^f,g^ |
| 21. | Sexual assault (rape, attempted rape, made to perform any type of sexual act through force or threat of harm)^f,g^ |
| 22. | Other unwanted or uncomfortable sexual experience^f,g^ |
| 23. | Combat or exposure to a war-zone (in the military or as a civilian)^f,g^ |
| 24. | Captivity (for example, being kidnapped, abducted, held hostage, prisoner of war)^f,g^ |
| 25. | Life-threatening illness or injury^f,g^ |
| 26. | Severe human suffering^f,g^ |
| 27. | Sudden violent death (for example, homicide, suicide)^f,g^ |
| 28. | Sudden accidental death^g^ |
| 29. | Serious injury, harm, or death you caused to someone else^f,g^ |
|  |  |
| Combat Experiences Scale | |
| 30. | Having to aid in the removal of unexploded land mines^h^ |
| 31. | Improvised explosive device/booby trap exploded near you^h^ |
| 32. | Patrolling areas (or riding in areas) where there were land mines^h^ |
| 33. | Being attacked or ambushed^h^ |
| 34. | Clearing/searching homes or buildings^h^ |
| 35. | Receiving small arms fire^h^ |
| 36. | Seeing dead bodies or body parts^h^ |
| 37. | Shooting or directing fire at the enemy^h^ |
| 38. | Being in threatening situations where you were unable to respond because of rules of engagement^h^ |
| 39. | Witnessing a friendly fire incident^h^ |
| 40. | Had a close call, dud landed near you^h^ |
| 41. | Provided aid to the wounded^h^ |
| 42. | Saved the life of a soldier or civilian^h^ |
| 43. | Handling or uncovering dead bodies or body parts^h^ |
| 44. | Witnessing violence within the local population or between ethnic groups^h^ |
| 45. | Witnessing an accident which resulted in serious injury or death^h^ |
| 46. | Seeing dead or seriously injured Americans^h^ |
| 47. | Had a close call, was shot or hit but protective gear saved you^h^ |
| 48. | Being wounded^h^ |
| 49. | Had a buddy shot or hit who was near you^h^ |
| 50. | Knowing someone seriously injured or killed^h^ |
| 51. | Having a member of your own unit become a casualty^h^ |
| 52. | Receiving incoming artillery rocket or mortar fire^h^ |
| 53. | Being directly responsible for the death of an enemy combatant^h^ |
| 54. | Being directly responsible for the death of a non-combatant^h^ |
| 55. | Successfully engaged the enemy^h^ |
| 56. | Being directly responsible for the death of a US or ally personnel^h^ |
| 57. | Calling in fire on the enemy^h^ |
| 58. | Engaging in hand to hand combat^h^ |
| 59. | Witnessing brutality/mistreatment toward non-combatants^h^ |
| 60. | Clearing/searching caves or bunkers^h^ |
|  |  |
| Deployment Experiences | |
| 61. | Being in an accident^i^ |
| 62. | Witnessing a suicide bombing^i^ |
| 63. | Having contact with traumatized civilians^i^ |
| 64. | Being shot at^i^ |
| 65. | During your most recent deployment, how often did you think you were in danger of being injured or killed^i^ |
| 66. | During your most recent deployment, did you handle any dead bodies^i^ |
|  |  |
| Life Experiences | |
| 67. | Serious physical assault (e.g., mugging), sexual assault, or rape^j,k^ |
| 68. | Serious assault happened to a close friend or relative^j,k^ |
| 69. | Murder of a close friend or relative^j,k^ |
| 70. | Suicide of a close friend or relative^j,k^ |
| 71. | Attempted suicide of a close friend or relative^j,k^ |
| 72. | Combat death of a close friend or relative^j,l^ |
| 73. | Accidental death of a close friend or relative^j,k^ |
| 74. | You witnessed someone being seriously injured or killed^j,k,l^ |
| 75. | You discovered or handled a dead body^j,k^ |
| 76. | You had a life-threatening illness or injury^j,l^ |
| 77. | You were in a disaster (e.g., hurricane, fire, flood, earthquake) where you could have died^j,k^ |
| 78. | You had any other experience that put you at risk of death or serious injury^j,k,l^ |
| 79. | You had a close loved one who had an experience that put them at risk of death or serious injury^j,k^ |
|  |  |
| Source | |
| ^a^ | Army STARRS New Soldier Study Questionnaire [7, 8] Available at: www.starrs-ls.org |
| ^b^ | Childhood Trauma Questionnaire (CTQ) [9, 10, 11] |
| ^c^ | Adverse Childhood Experiences (ACE) [12, 13] |
| ^d^ | Semi-Structured Assessment for Drug Dependence and Alcoholism (SSADDA) [14] |
| ^e^ | Posttraumatic Stress Disorder, diagnostic criterion A [15, 16] |
| ^f^ | Life Events Checklist (LEC) [17] |
| ^g^ | Life Events Checklist for DSM-5 (LEC-5) [18] |
| ^h^ | Combat Exposure Scale (CES) [19] |
| ^i^ | Developed for use in this study by the authors (QMB, RJU, CSF) |
| ^j^ | Army STARRS All Army Study Questionnaire [7, 8] Available at: www.starrs-ls.org |
| ^k^ | Deployment Risk and Resilience Inventory (DDRI) [20, 21] |
| ^l^ | Joint Mental Health Advisory Team 7 (J-MHAT 7) Operation Enduring Freedom 2010 Afghanistan |

***Daily assessments***

After completing the assessment of exposure to traumatic events and the assessment of PTSD, participants were instructed on completing the daily assessments and they selected the hour of the first daily assessment that best fit their daily routine. Participants were told to go about their normal daily activities during the daily diary assessment period and complete assessments at the specified times. Daily assessments were scheduled to start the next day. For the following 15 days, participants completed four daily assessments per day using an EMA methodology. In Phase 1 of the study, participants completed daily assessment on paper questionnaires. In Phase 2, participants completed the same questions on a portable electronic device (Apple Inc. iPad with software designed specifically for use in this study). Assessments were at fixed intervals, four hours apart (i.e., if the participant elected to start their first daily assessment at 8 a.m., then the second, third, and fourth daily assessments were at 12 p.m., 4 p.m., and 8 p.m., respectively). Participants were instructed to complete assessments within the first two hours when possible, but assessments were accepted for an additional four hours. All participants were provided the options of assessment alerts by text message, voice message, and a 3” x 2” x 1” portable alarm clock capable of 12 programmable alarms per day. Participants completing electronic assessments had the additional option of using alerts that were built into the electronic application. Participants using paper questionnaires recorded the date and time when each assessment was completed, and electronic assessments were automatically date and time stamped. Assessments not completed within the specified six-hour assessment period were dropped from data analysis.

***Assessment of sleep***

Sleep was assessed on the first daily assessment with the following 23 items.

How many hours of **actual sleep** did you get **last night**? (*This may be different than the number of hours you spent in bed.)* _______

How would you rate your sleep quality overall **last night**?

a. Very bad

b. Fairly bad

c. Fairly good

d. Very good

How many times did you wake up during the night **last night**? _______

Below is a list of sleep problems. Please fill in the bubble according to what you experienced **last night**.

|  | **No** | **Yes** |
| --- | --- | --- |
| *Trouble falling asleep* |  |  |
| Trouble falling asleep^a,b,c^ | ○ | ○ |
| Worried about being able to fall asleep^d^ | ○ | ○ |
| Worried about having disturbing dreams^d^ | ○ | ○ |
| *Somatic disturbance/sleep environment* |  |  |
| Had pain^b^ | ○ | ○ |
| Could not breathe comfortably^b^ | ○ | ○ |
| Coughed or snored loudly^b^ | ○ | ○ |
| Had to get up to use the bathroom^b^ | ○ | ○ |
| Felt too cold^b^ | ○ | ○ |
| Felt too hot^b^ | ○ | ○ |
| It was too noisy in my bedroom (i.e., partner or family member snored)^c^ | ○ | ○ |
| Had to get up because I was hungry or thirsty^d^ | ○ | ○ |
| *Parasomnia* |  |  |
| Performed an action during the night I do not remember (i.e., sleep walking)^c^ | ○ | ○ |
| Saw dreamlike images when falling asleep or waking up^c^ | ○ | ○ |
| Could not move when falling asleep or waking up (i.e., paralyzed)^c^ | ○ | ○ |
| *Difficulty staying asleep* |  |  |
| Trouble staying asleep^a,b^ | ○ | ○ |
| Repeated, disturbing dreams of a stressful experience^a^ | ○ | ○ |
| Had bad or frightening dreams^c^ | ○ | ○ |
| Distressing dreams that did **not** wake me up^d^ | ○ | ○ |
| Woke to a feeling of fear or terror^d^ | ○ | ○ |
| Woke up screaming or in a panic^d^ | ○ | ○ |

*Note*. Sleep items were adapted from the following sources: ^a^PTSD Checklist for the DSM-5 (PCL-5) [3], ^b^Pittsburgh Sleep Quality Index (PSQI) [22], ^c^SLEEP-50 [23], or ^d^developed for use in this study by the authors (RJU & CSF).

***Sleep problems: trouble falling asleep and difficulty staying asleep***

Sleep problems were assessed with 20 items adapted from the PCL-5 [3], PSQI [22], SLEEP-50 [23], or developed for use in this study by the authors (RJU & CSF). Item instructions were “Below is a list of sleep problems. Please fill in the bubble according to what you experienced last night.” And response choices were 0 (*No*) and 1 (*Yes*). For analysis, items were grouped into sleep problem dimensions based on existing sleep scale factors (e.g., sleep disturbances component in the PSQI) and the authors’ (QB, RJU, JW, GHW, & CSF) clinical consensus. Three items formed the dimension measuring trouble falling asleep, eight items formed the dimension measuring somatic disturbance/sleep environment, three items formed the dimension measuring parasomnia, and six items formed the dimension measuring difficulty staying asleep. A confirmatory factor analysis supported these dimensions [2]. The trouble falling asleep and difficulty staying asleep dimensions include items that are common sleep disturbances for individuals with PTSD [24, 25]. However, the somatic disturbance/sleep environment dimension is conceptually different from other sleep disturbances and the parasomnia dimension was so rarely endorsed that it could not be adequately measured, and both of these dimensions were dropped from further analyses.

**References**

[1] Biggs, Q. M., Ursano, R. J., Wang, J., Krantz, D. S., Carr, R. C., Wynn, G. H., Probe Adams, D., Dacuyan, N. M., & Fullerton, C. S. (2019). Daily variation in post traumatic stress symptoms in individuals with and without probable post traumatic stress disorder. *BMC Psychiatry, 19*:56. https://doi.org/10.1186/s12888-019-2041-7

[2] Biggs, Q. M., Ursano, R. J., Wang, J., Wynn, G. H., Carr, R. C., & Fullerton, C. S. (2020). Post traumatic stress symptom variation associated with sleep characteristics. *BMC Psychiatry, 20*:174. https://doi.org/10.1186/s12888-020-02550-y

[3] Weathers, F. W., Litz, B.T., Keane, T. M., Palmieri, P. A., Marx, B. P., & Schnurr, P. P. (2013). The PTSD Checklist for DSM-5 (PCL-5). Scale available from the National Center for PTSD at https://www.ptsd.va.gov

[4] Kroenke, K., Spitzer, R. L., & Williams, J. B. (2001). The PHQ-9: Validity of a brief depression severity measure. *Journal of General Internal Medicine, 16*, 606-613. https://doi.org/10.1046/j.1525-1497.2001.016009606.x

[5] Spitzer, R. L., Kroenke, K., & Williams, J. B. (1999). Validation and utility of a self-report version of PRIME-MD: the PHQ primary care study. Primary Care Evaluation of Mental Disorders. Patient Health Questionnaire. *JAMA, 282*, 1737-1744. https://doi.org/10.1001/jama.282.18.1737

[6] Spitzer, R. L., Kroenke, K., Williams, J. B., & Lowe, B. (2006). A brief measure for assessing generalized anxiety disorder: the GAD-7. *Archives of Internal Medicine, 166,* 1092-1097. https://doi.org/10.1001/archinte.166.10.1092

[7] Kessler, R. C., Colpe, L. J., Fullerton, C. S., Gebler, N., Naifeh, J. A., Nock, M. K., Sampson, N. A., Schoenbaum, M., Zaslavsky, A. M., Stein, M. B., Ursano, R. J., & Heeringa, S. G. (2013). Design of the Army Study to Assess Risk and Resilience in Service members (Army STARRS). *International Journal of Methods in Psychiatric Research, 22*(4), 267-275. https://doi.org/10.1002/mpr.1401

[8] Ursano, R. J., Colpe, L. J., Heeringa, S. G., Kessler, R. C., Schoenbaum, M., & Stein, M. B. (2014). The Army Study to Assess Risk and Resilience in Servicemembers (Army STARRS). *Psychiatry: Interpersonal and Biological Processes, 72*(2), 107-119. https://doi.org/10.1521/psyc.2014.77.2.107

[9] Bernstein, D. P., Ahluvalia, T., Pogge, D., & Handelsman, L. (1997). Validity of the Childhood Trauma Questionnaire in and adolescent psychiatric population. *Journal of the American Academy of Child & Adolescent Psychiatry, 36*(3), 340-348. https://doi.org/10.1097/00004583-199703000-00012

[10] Bernstein, D. P., Fink, F., Handelsman, L., Foote, J., Lovejoy, M., Wenzel, K., Sapareto, E., & Ruggiero, J. (1994). Initial reliability and validity of a new retrospective measure of child abuse and neglect. *American Journal of Psychiatry, 151*(8), 1132-1136. https://doi.org/10.1176/ajp.151.8.1132

[11] Bernstein, D. P., Stein, J. A., Newcomb, M. D., Walker, E., Pogge, D., Ahluvalia, T., Stokes, J., Handelsman, L., Medrano, M., Desmond, D., & Zule, W. (2003). Development and validation of a brief screening version of the Childhood Trauma Questionnaire. *Child Abuse & Neglect, 27*(2), 169-190. https://doi.org/10.1016/S0145-2134(02)00541-0

[12] Anda, R. F., Felitti, V. J., Bremner, J. D., Walker, J. D., Whitfield, C., Perry, B. D., Dube, S. R., & Giles, W. H. (2006). The enduring effects of abuse and related adverse experiences in childhood. A convergence of evidence from neurobiology and epidemiology. *European Archives of Psychiatry and Clinical Neuroscience, 256*(3), 174-186. https://doi.org/10.1007/s00406-005-0624-4

[13] Felitti, V. J., Anda, R. F., Nordenberg, D., Williamson, D. F., Spitz, A. M., Edwards, V., Koss, M. P., & Marks, J. S. (1998). Relationship of childhood abuse and household dysfunction to many of the leading causes of death in adults. The Adverse Childhood Experiences (ACE) Study. *American Journal of Preventive Medicine, 14*(4), 245-258. https://doi.org/10.1016/S0749-3797(98)00017-8

[14] Pierucci-Lagha, A., Gelernter, J., Feinn R., Cubells, J. F., Pearson, D., Pollastri, A., Farrer, L., & Kranzler, H. R. (2005). Diagnostic reliability of the Semi-structured Assessment for Drug Dependence and Alcoholism (SSADDA). *Drug and Alcohol Dependence, 80*(3)*,* 303-312. https://doi.org/10.1016/j.drugalcdep.2005.04.005

[15] American Psychiatric Association. (2013). *Diagnostic and statistical manual of mental disorders* (5th ed.). Arlington, VA: Author.

[16] American Psychiatric Association. (1994). *Diagnostic and statistical manual of mental disorders* (4th ed.). Washington, DC: Author.

[17] Gray, M. J., Litz, B. T., Hsu, J. L., & Lombardo, T. W. (2004). Psychometric properties of the Life Events Checklist. *Assessment, 11*(4), 330-341. https://doi.org/10.1177/1073191104269954

[18] Weathers, F. W., Blake, D. D., Schnurr, P. P., Kaloupek, D. G., Marx, B. P., & Keane, T. M. (2013). The Life Events Checklist for DSM-5 (LEC-5). Instrument available from the National Center for PTSD at https://www.ptsd.va.gov

[19] Killgore, W. D. S., Cotting, D. I., Thomas, J. L., Cox, A. L., McGurk, D., Vo, A. H., Castro, C. A., & Hoge, C. W. (2008). Post-combat invincibility: Violent combat experiences are associated with increased risk-taking propensity following deployment. *Journal of Psychiatric Research, 42*, 1112-1121. https://doi.org/10.1016/j.jpsychires.2008.01.001

[20] King, L. A., King, D. W., Vogt, D. S., Knight, J., & Samper, R. E. (2006). Deployment Risk and Resilience Inventory: A collection of measures for studying deployment-related experiences of military personnel and veterans. *Military Psychology, 18*(2), 89-120. https://doi.org/10.1207/s15327876mp1802_1

[21] Vogt, D. S., Proctor, S. P., King, D. W., King, L. A., & Vasterling, J. J. (2008). Validation of scales from the Deployment Risk and Resiliency Inventory in a sample of Operation Iraqi Freedom Veterans. *Assessment, 15*(4), 391-403. https://doi.org/10.1177/1073191108316030

[22] Buysse, D. J., Reynolds, 3rd C. F., Monk, T. H., Berman, S. R., & Kupfer, D. J. (1989). The Pittsburgh Sleep Quality Index: a new instrument for psychiatric practice and research. *Psychiatry Research, 28*, 193-213. https://doi.org/10.1016/0165-1781(89)90047-4

[23] Spoormaker, V. I., Verbeek, I., van den Bout, J., & Klip, E. C. (2005). Initial validation of the SLEEP-50 questionnaire. *Behavioral Sleep Medicine, 3*(4), 227-246. https://doi.org/10.1207/s15402010bsm0304_4

[24] McLay, R. N., Klam, W. P., & Volkert, S. L. (2010). Insomnia is the most commonly reported symptom and predicts other symptoms of post-traumatic stress disorder in U.S. Service members returning from military deployments. *Military Medicine, 175,* 759-762. https://doi.org/10.7205/milmed-d-10-00193

[25] Zayfert, C., & DeViva, J. C. (2004). Residual insomnia following cognitive behavioral therapy for PTSD. *Journal of Traumatic Stress, 17*, 69-73. https://doi.org/10.1023/B:JOTS.0000014679.31799.e7

**Supplement 2.** Table of Model Specification on Within-Subjects Residuals and Decomposition of Variance in the Total Sample and Participants with and without PTSD

|  | Total sample  (*N* = 157) | |  | With PTSD  (*n* = 80) | |  | Without PTSD  (*n* = 77) | |
| --- | --- | --- | --- | --- | --- | --- | --- | --- |
|  | CS^a^ | AR(1)^b^ |  | CS | AR(1) |  | CS | AR(1) |
| Sleep duration |  |  |  |  |  |  |  |  |
| Between-subjects variance | 1.479 | 1.435 |  | 1.448 | 1.358 |  | 1.338 | 1.321 |
| AR(1) | -- | 0.092 |  | -- | 0.144 |  | -- | 0.048 |
| Residual variance | 2.269 | 2.305 |  | 2.227 | 2.291 |  | 2.308 | 2.326 |
| ICC^c^ | 0.395 | 0.384 |  | 0.394 | 0.372 |  | 0.367 | 0.362 |
| Model fit statistics |  |  |  |  |  |  |  |  |
| -2LL | 7397.6 | 7384.7 |  | 3652.2 | 3637.6 |  | 3736.7 | 3734.9 |
| AIC | 7401.6 | 7390.7 |  | 3656.2 | 3643.6 |  | 3740.7 | 3740.9 |
| BIC | 7407.7 | 7399.9 |  | 3661.0 | 3650.7 |  | 3745.4 | 3747.9 |
| Sleep quality |  |  |  |  |  |  |  |  |
| Between-subjects variance | 0.186 | 0.176 |  | 0.162 | 0.144 |  | 0.191 | 0.189 |
| AR(1) | -- | 0.118 |  | -- | 0.208 |  | -- | 0.020 |
| Residual variance | 0.460 | 0.470 |  | 0.491 | 0.511 |  | 0.430 | 0.431 |
| ICC | 0.288 | 0.272 |  | 0.248 | 0.220 |  | 0.308 | 0.305 |
| Model fit statistics |  |  |  |  |  |  |  |  |
| -2LL | 4277.2 | 4257.4 |  | 2164.0 | 2133.7 |  | 2103.6 | 2103.3 |
| AIC | 4281.2 | 4263.4 |  | 2168.0 | 2139.7 |  | 2107.6 | 2109.3 |
| BIC | 4287.3 | 4272.5 |  | 2172.8 | 2146.8 |  | 2112.3 | 2116.4 |
| Trouble falling asleep |  |  |  |  |  |  |  |  |
| Between-subjects variance | 0.092 | 0.089 |  | 0.091 | 0.086 |  | 0.051 | 0.049 |
| AR(1) | -- | 0.251 |  | -- | 0.260 |  | -- | 0.242 |
| Residual variance | 0.054 | 0.056 |  | 0.064 | 0.068 |  | 0.043 | 0.045 |
| ICC | 0.630 | 0.614 |  | 0.587 | 0.558 |  | 0.543 | 0.521 |
| Model fit statistics |  |  |  |  |  |  |  |  |
| -2LL | 309.1 | 217.6 |  | 320.3 | 272.1 |  | -86.2 | -129.1 |
| AIC | 313.1 | 223.6 |  | 324.3 | 278.1 |  | -82.2 | -123.1 |
| BIC | 319.2 | 232.8 |  | 329.1 | 285.2 |  | -77.5 | -116.0 |
| Difficulty staying asleep |  |  |  |  |  |  |  |  |
| Between-subjects variance | 0.036 | 0.035 |  | 0.041 | 0.040 |  | 0.016 | 0.016 |
| AR(1) | -- | 0.094 |  | -- | 0.116 |  | -- | 0.060 |
| Residual variance | 0.037 | 0.038 |  | 0.047 | 0.048 |  | 0.028 | 0.028 |
| ICC | 0.493 | 0.479 |  | 0.466 | 0.455 |  | 0.364 | 0.364 |
| Model fit statistics |  |  |  |  |  |  |  |  |
| -2LL | -473.1 | -486.1 |  | -12.8 | -22.3 |  | -563.2 | -566.0 |
| AIC | -469.1 | -480.1 |  | -8.8 | -16.3 |  | -559.2 | -560.0 |
| BIC | -463.0 | -470.9 |  | -4.1 | -9.2 |  | -554.5 | -553.0 |

*Note*. -2LL = -2 log likelihood; AIC = Akaike information criterion; AR(1) = first-order autoregressive; BIC = Bayesian information criterion; CS = compound symmetry; ICC = intraclass correlation coefficient; PTSD = post traumatic stress disorder. ^a^The CS structure assumes the correlation between two assessments is constant regardless of their distance in time. ^b^The AR(1) structure assumes that the correlation between two assessments decreases exponentially as a function of the time interval between them. ^c^Intraclass correlation coefficient is calculated by the ratio of between-subjects variance by the total variance. For the total sample and individuals with PTSD, the model with AR(1) covariance structure provided a better fit to the data when compared to a CS structure (for example, change in -2 log likelihood for hours of sleep, -2LL = 7397.6-7384.7 = 12.9, *df* =1, *p* < .001). For individuals without PTSD, the model with AR(1) covariance structure did not provide a better fit to the data when compared to a CS structure for sleep duration, sleep quality, and trouble falling asleep. Results of DOW variation were essentially the same between CS and AR(1) covariance structure in individuals without PTSD. Thus, for consistency, AR(1) covariance structure was selected for all analyses.

**Supplement 3.** Table of Differences in Sleep Characteristics by the 7 Days of the Week in the Total Sample and Participants with and without PTSD

|  | Total sample  (*N* = 157) | |  | With PTSD  (*n* = 80) | |  | Without PTSD  (*n* = 77) | |
| --- | --- | --- | --- | --- | --- | --- | --- | --- |
|  | Coefficient  [95% CI] | *p* |  | Coefficient  [95% CI] | *p* |  | Coefficient  [95% CI] | *p* |
| Sleep duration |  |  |  |  |  |  |  |  |
| 7 DOW^a^ | *F*(6, 862) = 11.62 | <.001 |  | *F*(6, 431) = 5.08 | <.001 |  | *F*(6, 425) = 6.89 | <.001 |
| Monday | -0.69 [-0.94, -0.44] | <.001 |  | -0.55 [-0.90, -0.20] | .002 |  | -0.83 [-1.19, -0.48] | <.001 |
| Tuesday | -0.72 [-0.97, -0.47] | <.001 |  | -0.72 [-1.07, -0.36] | <.001 |  | -0.72 [-1.07, -0.36] | <.001 |
| Wednesday | -0.69 [-0.95, -0.44] | <.001 |  | -0.61 [-0.96, -0.25] | .001 |  | -0.77 [-1.13, -0.41] | <.001 |
| Thursday | -0.77 [-1.02, -0.53] | <.001 |  | -0.80 [-1.15, -0.45] | <.001 |  | -0.74 [-1.09, -0.40] | <.001 |
| Friday | -0.45 [-0.69, -0.22] | <.001 |  | -0.40 [-0.73, -0.07] | .017 |  | -0.49 [-0.83, -0.15] | .005 |
| Sunday | -0.11 [-0.35, 0.14] | .398 |  | -0.11 [-0.45, 0.23] | .536 |  | -0.10 [-0.45, 0.25] | .573 |
| Sleep quality |  |  |  |  |  |  |  |  |
| 7 DOW | *F*(6, 863) = 3.54 | .002 |  | *F*(6, 431) = 3.30 | .004 |  | *F*(6, 426) = 1.68 | .125 |
| Monday | -0.14 [-0.26, -0.03] | .013 |  | -0.10 [-0.27, 0.06] | .216 |  | -0.17 [-0.33, -0.02] | .030 |
| Tuesday | -0.19 [-0.30, -0.07] | .001 |  | -0.23 [-0.40, -0.06] | .007 |  | -0.12 [-0.28, 0.03] | .113 |
| Wednesday | -0.11 [-0.22, 0.01] | .066 |  | -0.08 [-0.25, 0.09] | .338 |  | -0.12 [-0.27, 0.04] | .136 |
| Thursday | -0.17 [-0.28, -0.06] | .003 |  | -0.25 [-0.41, -0.09] | .003 |  | -0.09 [-0.24, 0.06] | .250 |
| Friday | -0.02 [-0.12, 0.09] | .768 |  | -0.06 [-0.21, 0.09] | .456 |  | 0.03 [-0.12, 0.18] | .681 |
| Sunday | -0.03 [-0.14, 0.08] | .644 |  | 0.04 [-0.11, 0.19] | .614 |  | -0.08 [-0.24, 0.07] | .305 |
| Trouble falling asleep |  |  |  |  |  |  |  |  |
| 7 DOW | *F*(6, 863) = 3.01 | .007 |  | *F*(6, 431) = 2.95 | .008 |  | *F*(6, 426) = 1.28 | .263 |
| Monday | 0.05 [0.01, 0.08] | .019 |  | 0.06 [0.00, 0.11] | .065 |  | 0.04 [-0.01, 0.09] | .122 |
| Tuesday | 0.04 [0.00, 0.08] | .042 |  | 0.08 [0.02, 0.15] | .007 |  | 0.00 [-0.05, 0.05] | .972 |
| Wednesday | 0.03 [-0.01, 0.07] | .106 |  | 0.07 [0.01, 0.13] | .032 |  | 0.00 [-0.05, 0.05] | .974 |
| Thursday | 0.04 [0.00, 0.08] | .049 |  | 0.08 [0.02, 0.14] | .007 |  | 0.00 [-0.05, 0.04] | .886 |
| Friday | 0.03 [0.00, 0.07] | .050 |  | 0.06 [0.01, 0.12] | .019 |  | 0.01 [-0.04, 0.05] | .801 |
| Sunday | -0.02 [-0.06, 0.01] | .252 |  | -0.02 [-0.07, 0.04] | .524 |  | -0.02 [-0.07, 0.02] | .322 |
| Difficulty staying asleep |  |  |  |  |  |  |  |  |
| 7 DOW | *F*(6, 863) = 1.33 | .241 |  | *F*(6, 431) = 0.65 | .693 |  | *F*(6, 426) = 1.14 | .340 |
| Monday | 0.03 [0.00, 0.07] | .043 |  | 0.04 [-0.02, 0.09] | .172 |  | 0.03 [-0.01, 0.07] | .123 |
| Tuesday | 0.03 [-0.01, 0.06] | .114 |  | 0.04 [-0.01, 0.09] | .142 |  | 0.01 [-0.03, 0.05] | .508 |
| Wednesday | 0.02 [-0.01, 0.06] | .177 |  | 0.04 [-0.01, 0.09] | .157 |  | 0.01 [-0.03, 0.05] | .747 |
| Thursday | 0.01 [-0.02, 0.05] | .425 |  | 0.03 [-0.02, 0.08] | .225 |  | -0.01 [-0.04, 0.03] | .785 |
| Friday | 0.00 [-0.03, 0.03] | .836 |  | 0.01 [-0.04, 0.06] | .723 |  | -0.02 [-0.05, 0.02] | .403 |
| Sunday | 0.01 [-0.03, 0.04] | .687 |  | 0.02 [-0.03, 0.07] | .501 |  | 0.00 [-0.04, 0.03] | .815 |

*Note*. CI = confidence interval; DOW = day of week; PTSD = post traumatic stress disorder. Sex, age, race, education, and phase of the study were included as covariates. ^a^Saturday was set as the reference.

**Supplement 4a.** Table of Weekday versus Weekend Differences in Sleep Quality in the Total Sample and Participants with and without PTSD: Results of Mixed Models

|  | Total sample (*N* = 157) | |  | With PTSD (*N* = 80) | |  | Without PTSD (*N* = 77) | |
| --- | --- | --- | --- | --- | --- | --- | --- | --- |
| Parameter | Coefficient  [95% CI] | *p* |  | Coefficient  [95% CI] | *p* |  | Coefficient  [95% CI] | *p* |
| Fixed effects |  |  |  |  |  |  |  |  |
| Intercept | 1.76 [1.60, 1.93] | <.001 |  | 1.63 [1.44, 1.83] | <.001 |  | 1.72 [1.48, 1.95] | <.001 |
| Female vs. male | -0.01 [-0.16, 0.14] | .878 |  | -0.07 [-0.27, 0.13] | .498 |  | 0.05 [-0.20, 0.30] | .711 |
| Age^a^ | 0.00 [0.00, 0.01] | .199 |  | 0.01 [0.00, 0.02] | .017 |  | 0.00 [-0.01, 0.01] | .976 |
| White vs. non-white | -0.10 [-0.26, 0.06] | .221 |  | -0.23 [-0.44, -0.01] | .039 |  | 0.02 [-0.24, 0.27] | .890 |
| Some college or lower  vs. bachelor’s or higher^b^ | -0.08 [-0.25, 0.10] | .400 |  | 0.03 [-0.21, 0.28] | .780 |  | -0.14 [-0.42, 0.14] | .318 |
| Phase 1 vs. 2 | 0.01 [-0.17, 0.19] | .945 |  | 0.01 [-0.23, 0.26] | .920 |  | -0.04 [-0.32, 0.24] | .791 |
| Weekday vs. weekend | -0.10 [-0.17, -0.03] | .004 |  | -0.15 [-0.25, -0.04] | .007 |  | -0.05 [-0.15, 0.04] | .268 |
| PTSD vs. non-PTSD | -0.19 [-0.34, -0.04] | .012 |  | -- | -- |  | -- | -- |

*Note*. CI = confidence interval; PTSD = post traumatic stress disorder. ^a^Age was centered at the group mean. ^b^Bachelor’s degree or higher was set as the reference.

**Supplement 4b.** Table of Weekday versus Weekend Differences in Trouble Falling Asleep in the Total Sample and Participants with and without PTSD: Results of Mixed Models

|  | Total sample (*N* = 157) | |  | With PTSD (*N* = 80) | |  | Without PTSD (*N* = 77) | |
| --- | --- | --- | --- | --- | --- | --- | --- | --- |
| Parameter | Coefficient  [95% CI] | *p* |  | Coefficient  [95% CI] | *p* |  | Coefficient  [95% CI] | *p* |
| Fixed effects |  |  |  |  |  |  |  |  |
| Intercept | 0.18 [0.08, 0.27] | <.001 |  | 0.42 [0.29, 0.56] | <.001 |  | 0.20 [0.09, 0.31] | <.001 |
| Female vs. male | 0.08 [-0.01, 0.18] | .070 |  | 0.05 [-0.09, 0.19] | .474 |  | 0.11 [-0.01, 0.23] | .078 |
| Age^a^ | 0.00 [0.00, 0.00] | .721 |  | 0.00 [-0.01, 0.01] | .747 |  | 0.00 [0.00, 0.00] | .974 |
| White vs. non-white | -0.02 [-0.12, 0.07] | .618 |  | 0.00 [-0.15, 0.16] | .956 |  | -0.05 [-0.17, 0.07] | .431 |
| Some college or lower  vs. bachelor’s or higher^b^ | 0.01 [-0.09, 0.12] | .799 |  | 0.06 [-0.12, 0.24] | .504 |  | -0.02 [-0.15, 0.11] | .770 |
| Phase 1 vs. 2 | 0.02 [-0.08, 0.13] | .664 |  | -0.01 [-0.19, 0.16] | .888 |  | 0.05 [-0.08, 0.18] | .454 |
| Weekday vs. weekend | 0.05 [0.02, 0.07] | <.001 |  | 0.07 [0.04, 0.11] | <.001 |  | 0.02 [-0.01, 0.05] | .147 |
| PTSD vs. non-PTSD | 0.28 [0.19, 0.37] | <.001 |  | -- | -- |  | -- | -- |

*Note*. CI = confidence interval; PTSD = post traumatic stress disorder. ^a^Age was centered at the group mean. ^b^Bachelor’s degree or higher was set as the reference.

**Supplement 4c.** Table of Weekday versus Weekend Differences in Difficulty Staying Asleep in the Total Sample and Participants with and without PTSD: Results of Mixed Models

|  | Total sample (*N* = 157) | |  | With PTSD (*N* = 80) | |  | Without PTSD (*N* = 77) | |
| --- | --- | --- | --- | --- | --- | --- | --- | --- |
| Parameter | Coefficient  [95% CI] | *p* |  | Coefficient  [95% CI] | *p* |  | Coefficient  [95% CI] | *p* |
| Fixed effects |  |  |  |  |  |  |  |  |
| Intercept | 0.15 [0.09, 0.21] | <.001 |  | 0.29 [0.20, 0.38] | <.001 |  | 0.16 [0.10, 0.23] | <.001 |
| Female vs. male | 0.06 [0.01, 0.12] | .030 |  | 0.09 [-0.01, 0.18] | .065 |  | 0.03 [-0.04, 0.10] | .358 |
| Age^a^ | 0.00 [0.00, 0.00] | .701 |  | 0.00 [-0.01, 0.00] | .468 |  | 0.00 [0.00, 0.00] | .259 |
| White vs. non-white | -0.07 [-0.13, -0.01] | .024 |  | -0.09 [-0.19, 0.01] | .091 |  | -0.04 [-0.11, 0.03] | .238 |
| Some college or lower  vs. bachelor’s or higher^b^ | 0.06 [-0.01, 0.13] | .095 |  | 0.07 [-0.04, 0.19] | .215 |  | 0.01 [-0.07, 0.09] | .768 |
| Phase 1 vs. 2 | -0.04 [-0.10, 0.03] | .302 |  | -0.05 [-0.16, 0.06] | .395 |  | -0.01 [-0.09, 0.07] | .845 |
| Weekday vs. weekend | 0.01 [-0.01, 0.03] | .156 |  | 0.02 [-0.01, 0.05] | .205 |  | 0.01 [-0.02, 0.03] | .536 |
| PTSD vs. non-PTSD | 0.16 [0.10, 0.22] | <.001 |  | -- | -- |  | -- | -- |

*Note*. CI = confidence interval; PTSD = post traumatic stress disorder. ^a^Age was centered at the group mean. ^b^Bachelor’s degree or higher was set as the reference.
